# Proteostasis of Huntingtin in Health and Disease

**DOI:** 10.3390/ijms18071568

**Published:** 2017-07-19

**Authors:** Seda Koyuncu, Azra Fatima, Ricardo Gutierrez-Garcia, David Vilchez

**Affiliations:** Cologne Excellence Cluster for Cellular Stress Responses in Aging-Associated Diseases (CECAD), University of Cologne, Joseph Stelzmann Strasse 26, 50931 Cologne, Germany; skoyunc2@uni-koeln.de (S.K.); afatima@uni-koeln.de (A.F.); rgutier2@smail.uni-koeln.de (R.G.-G.)

**Keywords:** autophagy, aging, chaperones, Huntington’s disease, neurodegeneration, proteostasis, proteasome

## Abstract

Huntington’s disease (HD) is a fatal neurodegenerative disorder characterized by motor dysfunction, cognitive deficits and psychosis. HD is caused by mutations in the *Huntingtin* (*HTT*) gene, resulting in the expansion of polyglutamine (polyQ) repeats in the HTT protein. Mutant HTT is prone to aggregation, and the accumulation of polyQ-expanded fibrils as well as intermediate oligomers formed during the aggregation process contribute to neurodegeneration. Distinct protein homeostasis (proteostasis) nodes such as chaperone-mediated folding and proteolytic systems regulate the aggregation and degradation of HTT. Moreover, polyQ-expanded HTT fibrils and oligomers can lead to a global collapse in neuronal proteostasis, a process that contributes to neurodegeneration. The ability to maintain proteostasis of HTT declines during the aging process. Conversely, mechanisms that preserve proteostasis delay the onset of HD. Here we will review the link between proteostasis, aging and HD-related changes.

## 1. Introduction

**Figure 1:**
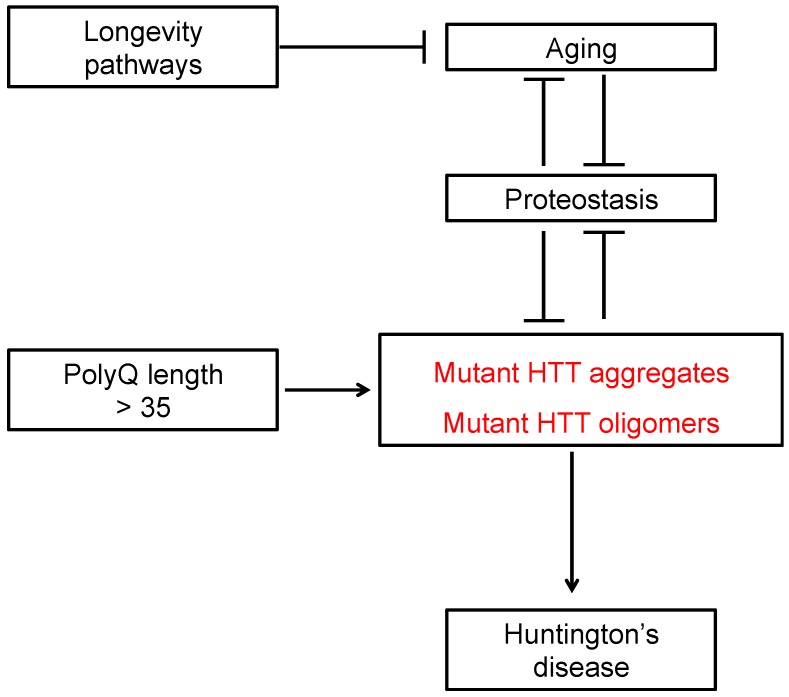
Huntington’s disease (HD) is caused by mutations in the *Huntingtin* gene (*HTT*) that expand the polyQ stretch of HTT protein, resulting in its aggregation and proteotoxicity. In individuals affected by HD, HTT contains >35 polyQ repeats. The length of the polyQ stretch correlates with the accumulation of toxic polyQ-expanded HTT aggregates and oligomers. The protein homeostasis (proteostasis) network controls the folding and clearance of mutant HTT, reducing its proteotoxicity. However, the accumulation of polyQ-expanded HTT aggregates and oligomers can result in a collapse of proteostasis, a feature that contributes to the neurodegeneration phenotype. With age, organisms lose the ability to maintain the proteostasis network; a process that could explain the late onset of HD. Conversely, mechanisms that extend longevity sustain proteostasis with age, reducing the accumulation of toxic mutant HTT aggregates and oligomers.

**Figure 2:**
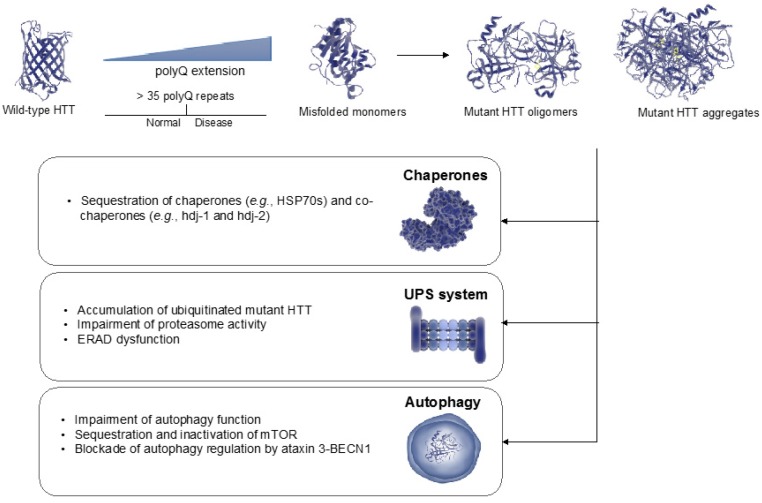
Proteostasis collapse induced by mutant HTT. When HTT contains more than 35 polyQ repeats, it aggregates and also forms toxic oligomers. The accumulation of polyQ-expanded aggregates and oligomers collapses distinct proteostasis nodes such as the chaperone network, the ubiquitin-proteasome system (UPS), endoplasmic reticulum-associated degradation (ERAD) and autophagy. Moreover, wild-type HTT act as a scaffolding protein directly involved in autophagy regulation whereas mutations in HTT can disrupt its normal function.

Huntington’s disease (HD) is the most common inherited neurodegenerative disorder with an incidence of 0.38 per 100,000 persons per year [1,2]. The characteristic symptoms of HD are excessive motor movements, cognitive decline and psychiatric abnormalities [3,4]. Neuronal loss occurs in many brain regions, but striatal medium spiny neurons undergo the greatest neurodegeneration [5,6]. HD patients usually die within 15–20 years of the disease onset [4]. HD is inherited in a dominant manner and caused by abnormal expansions of CAG repeats in the *Huntingtin* (*HTT*) gene [1]. These mutations expand the polyglutamine stretch (polyQ) of the *N*-terminal domain of the HTT protein, resulting in proteotoxicity and protein aggregation [1,7,8]. HTT is variable in its structure, as the many polymorphisms of the gene lead to different numbers of polyQ repeats in the protein. The wild-type *HTT* gene encodes a large protein of approximately 350 kDa that contains 6–35 polyQ repeats. In individuals affected by HD, HTT protein contains >35 polyQ repeats [1]. Normal function of HTT is involved in several biological processes such as transcriptional regulation, ciliogenesis, apoptosis, vesicle trafficking, autophagy and embryonic development [9]. Although loss of normal function could also contribute to HD [9,10], experimental data and the dominant inheritance pattern of HD indicate that gain-of-function of mutant HTT is toxic and triggers neurodegeneration (Figure 1) [1,11,12,13]. The function, folding and clearance of HTT are controlled by different protein homeostasis (proteostasis) mechanisms including the chaperome network, the ubiquitin-proteasome system (UPS) and autophagy-lysosome pathways [14,15,16,17,18]. The accumulation of polyQ-expanded aggregates has been proposed to contribute to neurodegeneration [19]. However, the molecular mechanisms by which these aggregates induce neural dysfunction and death remain unsolved. PolyQ-expanded fibrils may collapse distinct proteostasis nodes such as chaperones and protein clearance mechanisms (Figure 2) [20,21,22,23], sequester regulatory components such as transcription factors [24] or physically obstruct neuronal extensions [25]. Growing evidence indicates that intermediate species called “oligomers” formed during the aggregation or disaggregation process also induce neurotoxicity [26,27,28,29].

**Figure 3:**
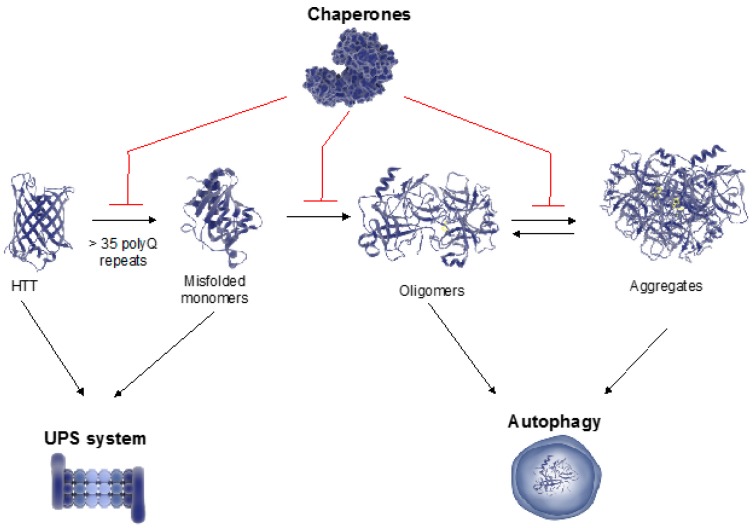
The proteostasis network reduces aggregation and proteotoxicity of mutant HTT. Different proteostasis nodes such as the chaperome network, the UPS and autophagy-lysosome pathways, control the folding, aggregation and clearance of HTT. Chaperones participate at distinct levels of HTT proteostasis, including the regulation of aggregation and disaggregation processes to reduce the accumulation of aberrant inclusions and/or intermediates. If refolding activities are not sufficient to assure the proper function of HTT, chaperones may also promote its degradation through protein clearance mechanisms. The UPS is mostly involved in the degradation of HTT monomers whereas autophagy can degrade large polyQ-expanded HTT aggregates.

Whereas mutant HTT can collapse the proteostasis network, mechanisms that preserve or increase proteostasis ameliorate HD-related changes (Figure 1, Figure 2 and Figure 3) [17]. Conversely, a decline in the ability to maintain proteostasis during the aging process could underlie the late onset of HD (Figure 1). In this regard, it is important to note that the length of the polyQ repeats correlates with the disease progression and unusually long polyQ stretches predict younger HD onset [1,30,31]. Experiments in model organisms show that highly expanded polyQ repeats accelerates HD-related changes, proteotoxicity and protein aggregation [17,32], suggesting that the cellular ability to maintain proteostasis of mutant HTT depends on the polyQ length. In this review, we discuss recent insights into distinct nodes of proteostasis of HTT and how this network can be adapted to ameliorate HD.

## 2. Regulation of Mutant HTT Aggregation by the Chaperome Network

Nascent proteins are synthesized by the ribosome as linear chains of amino acids that must fold into the correct three-dimensional structure to perform their biological function(s) [33]. This process is particularly inefficient for larger proteins and its dysfunction can lead to protein aggregation [33]. Molecular chaperones facilitate the folding of newly synthesized proteins into their proper native conformation [33,34,35,36]. Moreover, chaperones assure the proper folding and localization of proteins during all their life cycle. When the structure of proteins is challenged by stress conditions or aggregation-prone mutations (e.g., polyQ-expanded HTT), a series of cellular stress responses are activated to correct proteostatic deficiencies [37,38]. For instance, chaperones assist the refolding of proteins when their structure is challenged upon stress conditions such as hyperthermia, hypoxia, oxidative stress, exposure to toxins and misfolding-prone mutations [36,37,39,40]. Moreover, chaperones bind to exposed hydrophobic regions of nascent or misfolded polypeptides, thus preventing these residues from forming aberrant interactions with other proteins [41]. In addition, chaperones participate at different levels of protein aggregation and disaggregation processes to reduce aberrant inclusions or intermediates [34]. If refolding activities are not sufficient to assure the proper function of proteins, chaperones promote the degradation of damaged and misfolded proteins [37]. Thus, the chaperome network (i.e., chaperones and co-chaperones) preserves proteostasis by preventing protein misfolding and aggregation.

The human chaperome network consists of 88 chaperones and 244 co-chaperones [42]. Chaperones are classified according to their molecular weight into six conserved classes: 40 kilodalton heat shock proteins (HSP40s), HSP60s (chaperonins), HSP70s, HSP90s, HSP100, and the small HSPs [43]. Chaperones are also subdivided into two groups depending on their source of energy for the chaperone activity: ATP-dependent (e.g., HSP70s, HSP90s, HSP100s, chaperonins) and ATP-independent molecular chaperones (e.g., small HSPs and conditionally activated chaperones) [42,44,45]. Whereas distinct chaperones such as members of the HSP90 and HSP70 families are involved in different aspects of the proteostasis network (e.g., folding of nascent proteins, refolding, degradation of unnecessary or damaged proteins), specific set of chaperones evolved to primarily protect the cell from proteotoxic stress [46]. Multiple co-chaperones assist chaperones in their vast range of proteostatic tasks such as the tetratricopeptide repeat (TPR)-domain-containing family (e.g., CHIP, HOP), the BAG-domain-containing family (BAG1–BAG6) and DNAJ-domain-containing HSP40s [42,43].

Defects in chaperone function are linked with HD [37,47,48]. Indeed, numerous studies in HD mammalian cells and model organisms (e.g., yeast, worms, files and mice) have highlighted the direct role of chaperones in modulating mutant HTT aggregation and toxicity (Figure 3) [42,47,49,50,51,52]. Distinct molecular chaperones can modulate polyQ-expanded toxicity through different mechanisms, showing synergy in suppression of neurodegeneration [49]. For instance, chaperones can inhibit polyQ-expanded aggregation by either preventing intramolecular conformational changes in mutant HTT or promoting its degradation via protein clearance mechanisms. Chaperones can also interfere at different steps of the aggregation process such as primary nucleation, elongation and fragmentation of fibril and secondary nucleation [34].

The interaction of members of the Hsp70 family and their DNAJ-domain-containing Hsp40 co-chaperones (e.g., hdj-1) inhibit the formation of spherical aggregates by promoting the accumulation of less toxic fibrillar and amorphous inclusions [53,54]. Overexpression of Hsp40s and/or Hsp70s in *D. melanogaster* and mouse models suppresses polyQ-mediated neurodegeneration [55,56,57,58,59]. Besides multiple Hsp40s [42,51,57,60,61], other Hsp70s co-chaperones also determine polyQ-expanded aggregation and toxicity. For instance, the co-chaperones BAG1 and CHIP are involved in the aggregation fate of mutant HTT via their interaction with HSP70s [62,63,64]. The differential ability to induce the HSP70 system exhibited by different neuronal types could provide a potential explanation to the higher neurodegeneration of the striatum in HD [65]. Notably, cerebellar neurons induce Hsp70s levels upon mutant HTT overexpression. On the contrary, striatal neurons cannot sufficiently upregulate their chaperone system to overcome this proteotoxic stress [65].

Whereas numerous independent studies established a link between HSP70s and HD, in vitro experiments indicate that HSP90s do not directly modulate polyQ aggregation [54]. Indeed, overexpression of members of the HSP90 family does not suppress polyQ toxicity [51]. However, the inhibition of HSP90 induces heat-shock response (HSR) and reduces polyQ-expanded HTT aggregation in HD organismal models and cells [66]. The HSR is a cellular mechanism controlled by the transcription factor HSF1 that induces the expression of multiple chaperones to ameliorate acute proteotoxic stress [67]. Interestingly, activation of HSR delays HD-related changes in *D. melanogaster* and *C. elegans* models [59,68,69]. However, the beneficial effects of HSR activation in HD mouse models were transient and diminished with disease progression [70]. Moreover, the transgenic overexpression of HSR-inducible Hsp70 (HSPA1A) and the small Hsp27 (HSPB1) in mouse models is characterized by modest or absent effects in HD onset and aggregate formation [71,72,73]. Although mutant HTT induces a mild upregulation of several small HSPs (e.g., Hspb3, Hspb6, Hspb7) [74], the expression of polyQ-expanded proteins per se does not activate the HSR, providing a potential explanation to the lack of strong effects of HSR-canonical chaperones in polyQ disease [75]. In this regard, a screen for polyQ modifiers identified two DNAJ-containing HSP40s (DNAJB6 and DNAJB8), which are not activated by HSF1 [76]. Remarkably, DNAJB6 inhibits the conversion of soluble polyQ-expanded HTT peptides into amyloid fibrils, particularly by suppressing primary nucleation of aggregation [77]. Moreover, DNAJB6 overexpression delays polyQ-expanded aggregation and extends lifespan in HD mouse models [77].

Another key regulator of mutant HTT aggregation is the TRiC/CCT complex (T-complex protein-1 ring complex, also called CCT for chaperonin containing TCP1). This chaperonin complex consists of two stacked rings formed by eight paralogous subunits (CCT1–CCT8) [78,79]. Mutation or loss of a single subunit is sufficient to impair the assembly and function of the TRiC/CCT complex [79], a process that accelerates mutant HTT aggregation and worsens HD-related changes [42,50,51,52]. Conversely, simultaneous overexpression of all eight CCT subunits reduces polyQ-expanded HTT aggregation and neurodegeneration [50]. Ectopic expression of specific single subunits (i.e., CCT1, CCT8) can also ameliorate HD-related changes. Since CCT8 is sufficient to increase the assembly of the TRiC/CCT complex, overexpression of this single subunit results in reduced polyQ aggregation [80]. Although CCT1 overexpression does not increase TRiC/CCT assembly, this subunit promotes the interaction between HTT and TRiC/CCT complex resulting in a remodeling of HTT aggregates and, therefore, reduced neurotoxicity [52].

Taken together, the chaperome network is a central modulator of mutant HTT aggregation and toxicity. In addition, it is also important to note that polyQ aggregates can also sequester molecular chaperones such as inducible and constitutively expressed HSP70s and their co-chaperones (e.g., hdj-1, hdj-2), a process that could directly contribute to the proteostasis collapse and neurodegeneration observed in HD (Figure 2) [7,24,81,82,83].

## 3. The Mechanistic Links between the Ubiquitin Proteasome-System (UPS) and HD

Protein clearance mechanisms are essential to adjust the proteome composition to the specific requirements of a particular cell type and status [17,84], including the degradation of numerous cellular regulatory factors and structural components. In addition, protein clearance systems terminate toxic, damaged and misfolded proteins to diminish aberrant protein aggregation and proteotoxicity [17,84]. Impairment of protein clearance mechanisms contributes to HD pathology [85,86,87]. Conversely, enhancement of these mechanisms can ameliorate the proteotoxic stress associated with polyQ-expanded aggregation [15,17,88]. Notably, blockade of mutant HTT expression in inducible HD models at an age that have already presented symptomatic changes results in disappearance of pathological aggregates and amelioration of behavioral phenotype [89]. These findings provided experimental evidence of clearance mechanisms to scavenge polyQ-expanded HTT aggregates in neurons.

**Figure 4:**
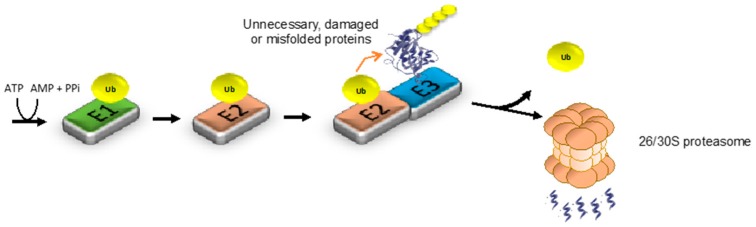
The ubiquitin-proteasome system (UPS). Unnecessary, damaged and misfolded proteins are marked for proteasomal degradation by the attachment of ubiquitin molecules via a three-step sequential mechanism. In the first step, ubiquitin is activated in an ATP-dependent manner by the ubiquitin-activating enzyme (E1). Then, activated ubiquitin is transferred to ubiquitin conjugating enzymes (E2s) forming E2-ubiquitin thioester structures. Finally, E3 ligases catalyze the attachment of ubiquitin to their specific substrate by binding both the E2-ubiquitin thioester structure and the target protein. The same cascade ubiquitination mechanism links additional molecules to the primary ubiquitin through internal ubiquitin lysines, forming a polyubiquitin chain. A chain of at least four lysine 48-linked ubiquitins is considered the primary signal for proteasomal degradation. E3 enzymes confer specificity to the UPS. Accordingly, more than 600 E3 ligases have been identified in humans so far. After the polyubiquitination cascade process, target substrates are recognized and degraded by the 26S/30S proteasome.

The ubiquitin proteasome-system (UPS) is the primary selective proteolytic process, regulating the degradation of both unnecessary and misfolded proteins [17]. Proteins are tagged for proteasomal degradation by the attachment of ubiquitin molecules, an evolutionary conserved small protein of 8.5 kDa (Figure 4) [90]. Ubiquitination is accomplished through a sequential mechanism involving three enzymes [91]. In the first step of the ubiquitination cascade, the E1 ubiquitin-activating enzyme activates ubiquitin in an ATP-dependent manner. Then, activated ubiquitin is transferred to an E2 ubiquitin-conjugating enzyme. In the third step, E3 ubiquitin ligases catalyze the transfer of ubiquitin to their specific target proteins [92]. The same three-step sequential mechanism links additional ubiquitin molecules to one of the seven lysine residues of the primary ubiquitin, forming a polyubiquitin chain. A chain of at least four lysine 48-linked ubiquitin molecules is the main signal for proteasomal recognition and degradation, whereas other ubiquitin chains participate in different biological processes such as signal transduction [93]. The proteasome is a macromolecular proteolytic machinery formed by the assembly of multiple subunits. The main core of the proteasome is the 20S particle, which contains the catalytic subunits [94]. However, free 20S complex is normally inactive in the cell and its activation requires the binding of regulatory proteasome particles [95]. The most common regulatory particle is the 19S core, which binds to 20S forming active 26S/30S proteasomes [96,97,98]. 19S is central for recognition, unfolding and, finally, translocation to the catalytic 20S core of polyubiquitinated targets [17,97,98]. Besides the 19S, other regulatory factors can also activate the 20S catalytic core such as the Blm10/PA200 protein or the PA28 complex (also known as 11S) [99].

Cumulative evidence indicates a role of the UPS in the regulation of polyQ-expanded HTT aggregation and toxicity (Figure 3). In human HD postmortem brains and model organisms, polyQ-expanded HTT inclusions contain ubiquitinated proteins as well as components of the proteasome [24,100,101,102,103,104,105]. Moreover, pharmacological studies based on both HD cellular and animal models showed that downregulation of proteasome activity by specific inhibitors results in increased aggregation of mutant HTT [105,106]. Likewise, knockdown of different proteasome subunits results in diminished proteasome activity and triggers the accumulation of polyQ-expanded repeats in HD models [51,86]. Conversely, enhancement of proteasome assembly/activity has been proven beneficial in HD organismal models. For instance, proteasome activation with sulforaphane promotes elimination of mutant HTT in cell culture [107]. Overexpression of specific proteasome subunits also ameliorates HD-related changes. For example, the scaffolding proteasome subunit PSMD11/Rpn6 promotes the assembly of 26S/30S proteasomes and decreases polyQ aggregation in HD *C. elegans* models [86]. A genetic gain-of-function screen showed that increased levels of Rpn11 block the age-associated decline of 26S/30S proteasome activity, suppressing polyQ-expanded aggregation and neurodegeneration in HD *D. melanogaster* models [108]. Increased expression of other proteasome regulators such as PA28γ activator also ameliorates neurodegeneration induced by polyQ-expanded HTT in striatal neuronal cultures [109]. Besides direct proteasome activity regulators, overexpression of other UPS components also has beneficial effects in HD models. For instance, ectopic expression of ubiquilin-1, a factor that facilitates protein disposal through the proteasome, delays mutant HTT accumulation and extends lifespan in HD mouse models [110]. Moreover, the overexpression of distinct proteins with E3 ubiquitin ligase activity (e.g., Hrd1, Parkin, CHIP) improves clearance of mutant HTT and reduces cell death [62,111,112].

Besides the direct role of the UPS in reducing the accumulation of mutant HTT oligomers and aggregates, the impairment of proteasome activity by polyQ-expanded HTT also determines the severity of HD-related changes [113] (Figure 2). In contrast to the soluble form of HTT, aggregated HTT has been found ubiquitinated itself, suggesting an impairment of the UPS to degrade polyQ-expanded HTT that could aggravate HD [101]. Moreover, proteasome activity is downregulated in HD patients and mice models [101]. These findings support the hypothesis of a global dysfunction of proteasome function induced by mutant HTT, a process that could result in proteostasis collapse [20]. Indeed, in vitro studies suggest that the proteasome is not able to cleave within expanded polyQ stretches and the failure of these undegradable sequences to exit the proteasome could affect proteasome activity [114]. Notably, cells transiently transfected with polyQ-expanded fragments exhibit decrease proteasome activity [115]. Likewise, degradation of proteasomal reporters (i.e., ubiquitinated GFP) is impaired in cells overexpressing a fragment of mutant HTT [22,116,117]. In HD mouse models, the expression of ubiquitinated mutant HTT in its aggregated form inhibits 26S/30S proteasome activity [21]. A potential molecular mechanism to explain this phenomenon could rely on intrinsic properties of polyQ proteins that could prevent their efficient clearance, resulting in the trapping of proteasomes within aggregates [22]. Although these findings suggest that mutant HTT impairs the proteasome machinery, it is important to note that other studies do not support this model. For instance, a study reported increased proteasome activity in neuronal cells expressing polyQ-expanded HTT [102]. In addition, other studies did not find a direct effect of polyQ aggregates on blocking 26S/30S proteasomes [116,117], suggesting that mutant HTT could alter UPS function by impinging on either the activity or distribution of UPS modulators. Alternatively, alteration of proteasome activity could also be due to a general proteostasis collapse induced by the sequestration of chaperones by mutant HTT and the concomitant accumulation of misfolded proteins [117]. Interestingly, mutant HTT not only affects proteasomal degradation of cytosolic proteins but also ER-associated degradation (ERAD), a process that induces ER stress [27]. Remarkably, ERAD collapse is triggered by the sequestration of the cytosolic chaperone p97/VCP [27]. Since ER stress precedes the accumulation of polyQ aggregates, the presence of oligomeric mutant HTT could be sufficient to induce sequestration of p97/VCP chaperone [27]. Altogether, the findings discussed here indicate that: (1) the proteasome directly contributes to clear mutant HTT and inhibit its aggregation; and (2) proteasome activity is impaired by mutant HTT, a process that could contribute to general proteostasis collapse and neurodegeneration. Thus, modulation of the UPS could be a potential therapeutic approach for HD.

## 4. The Dual Role of PolyQ-Expanded HTT in the Autophagy-Lysosome System

**Figure 5:**
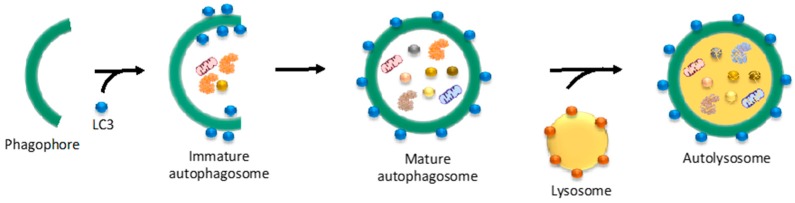
The autophagy-lysosome system. Autophagy, or macroautophagy, starts with the formation of a double membrane structure known as the phagophore, which can be either newly synthesized or originated from the plasma membrane, ER or mitochondria. The ULK complex (formed by, among others, ULK1/2, ATG13, FIP200 and ATG101) regulates this first step of autophagy. Then, the VPS34-BECN1 complex (formed by VPS34, BECN1, AMBRA1, ATG14L and other proteins) promotes the expansion of the phagophore. Once the cytoplasmic fraction is engulfed into the phagophore, the membrane elongates until it closes forming the autophagosome, a process regulated by the ATG12-ATG5-ATG16L1 complex. Conjugation of cytosolic LC3 (LC3I) to phosphatidylethanolamine generates LC3II, which is recruited to the membrane of the autophagosome. Finally, LC3II-containing autophagosomes are trafficked to the lysosome for degradation of their content including polyQ-expanded aggregates.

Autophagy degrades cytosolic fractions as well as damaged or outlived organelles and macromolecules, including proteins. Autophagy not only function as a cellular recycling system but also as a protective mechanism to terminate misfolded and aggregated proteins [17,37]. The catalytic component of autophagy is the lysosome, a membrane-bound organelle that contains cellular hydrolases, nucleotidases, glycosidases, lipases, and proteases. The three most characterized types of autophagy are macroautophagy, microautophagy and chaperone-mediated autophagy (CMA), which differ on their cargo recognition and delivery system to lysosomes [118]. Macroautophagy (hereafter referred as autophagy) is a bulk clearance process that starts with the formation of a double membrane structure known as the phagophore, a process regulated by the ULK complex (Figure 5) [84,119,120]. Then, the VPS34-BECN1 complex promotes the expansion of the phagophore. Once the cytoplasmic fraction and/or organelles are engulfed into the phagophore, the membrane elongates until it closes forming the autophagosome. Finally, autophagosomes are trafficked and fused to the lysosome for degradation of their content. In microautophagy, parts of the cytoplasm are delivered directly to the lysosomes [121]. In CMA, chaperones such as HSP70 mediate the degradation of proteins with the pentapeptide motif KFERQ [122].

Whereas autophagy and microautophagy can degrade organelles and proteins, CMA is essentially involved in protein degradation [123]. Although autophagy was originally defined as a bulk degradation mechanism, it is emerging as a selective proteolytic system where adapters/receptors such as p62/SQSTM1 and NBR1 bind to both ubiquitinated proteins and autophagy components to induce lysosomal degradation of specific cargos [85,124,125]. The discovery of these adapters provided a molecular link between autophagy and the UPS, which could lead to novel therapeutic strategies in proteostasis-related diseases [124]. In this regard, it is important to note that autophagy is able to degrade large protein complexes and aggregates [17,37], whereas protein inclusions block the proteasome machinery (Figure 2 and Figure 3) [116]. This role of autophagy in proteostasis includes degradation of aberrant aggregates triggered by polyQ-expanded HTT expression [126,127]. Accordingly, dysregulation of autophagy hastens HD-related changes. For instance, loss of p62/SQTM1 increases cell death induced by mutant HTT [128]. Conversely, enhancement of autophagy ameliorates HD-related proteotoxicity [88,129,130]. For example, inhibition of mTOR by rapamycin induces autophagy resulting in decreased toxicity of mutant HTT in flies and mice [88]. Likewise, small molecules that activate autophagy promote clearance of polyQ-expanded HTT in yeast, fly and mammalian HD models [130].

Besides the role of autophagy in the degradation of mutant HTT aggregates, several independent findings reported autophagy dysfunction in HD [131]. For instance, HD mouse models and cells from HD patients exhibit impaired ability of autophagic vacuoles to recognize cytosolic cargo [132]. Remarkably, HTT can function as a scaffolding protein for selective autophagy, having a direct role on autophagy regulation [133]. Thus, HTT has a dual impact on HD as an aggregation-prone protein and also as a scaffolding protein involved in autophagy, adding a new layer of complexity to HD [133]. Whereas the *N*-terminal domain of HTT contains the polyQ-expanded region that leads to its aggregation, the *C*-terminal domain is essential to bind to ULK1 and p62. Interaction of HTT with these factors is crucial for the progress of two main stages of autophagy: autophagy induction and cargo recognition [133]. Moreover, the *C*-terminal domain of mutant HTT interacts with p97/VCP in the mitochondria inducing mitophagy, a selective form of autophagy that degrades this organelle [134]. In parallel, the *N*-terminal domain also participates in activation of autophagy as mutant HTT aggregates sequester and inactivate mTOR [88]. Moreover, HTT can undergo proteolytic post-translational modifications by caspases that generate *N*- and *C*-terminal fragments [135]. The addition of 14 carbon myristate to a glycine residue exposed on a caspase-3-cleaved 34-amino acid fragment of HTT (HTT_553–586_) induces autophagosome formation, a process altered in HD cellular models [136,137]. Notably, it has been recently shown that the isolated polyQ tract of the deubiquitinating enzyme ataxin 3 is sufficient to regulate BECN1 and autophagy in an mTOR-independent manner. However, a polyQ-expanded *N*-terminal HTT fragment comprising exon 1, which occurs in vivo as a result of alternative splicing, blocks ataxin 3-BECN1 interaction in a competing manner resulting in impaired autophagy [138]. Taken together, these findings highlight the key role of autophagy in HD, either as a mechanism to scavenge mutant HTT or an essential biological process directly regulated by HTT function (Figure 2 and Figure 3).

## 5. The Melding Fields of Proteostasis of Aging, Pluripotency and HD

With age, the proteostasis network undergoes a decline in its function, a process that contributes to the onset of age-related diseases such as HD (Figure 1) [139]. For instance, the expression of 32% of the chaperome network is downregulated in human brains with age [42]. These include ATP-dependent chaperones such as cytosolic HSP90, HSP70 family members (e.g., HSPA8, HSPA14) and subunits of the TRiC/CCT complex [42]. On the other hand, only 19.5% of the chaperome network is upregulated during human brain aging [42]. Interestingly, these changes in the chaperome network were further promoted in the brains of HD patients when compared to age-matched control groups [42]. In support of a role of chaperone dysfunction in HD, knockdown experiments of distinct chaperones which are downregulated during aging (e.g., HSPA14, HSPA8 or CCT subunits) worsen proteotoxicity in HD mammalian and *C*. *elegans* models [42]. Likewise, protein clearance mechanisms are downregulated with aging and mimicking this condition at early ages hastens HD-related changes in disease models [17]. A direct link between aging, polyQ-expanded aggregation and HD-related changes has been established in invertebrate models [32,140,141]. In both *D. melanogaster* and *C. elegans* models that express polyQ of different lengths, the onset of polyQ-expanded aggregation and severity of neurodegeneration not only correlates with repeat length but also age [32,140,141]. Concomitantly, the proteostasis collapse induced by polyQ aggregates in these models [141,142] could further accelerate age-related proteostasis and the aging process itself. Indeed, treating *C. elegans* models with Thioflavin T, a compound that binds polyQ-expanded aggregates, slows protein aggregation and dramatically extends lifespan [143,144]. Remarkably, mechanisms that extend longevity enhance the proteostasis network and preserve the integrity of the proteome during aging [17,48]. For instance, well-characterized longevity mechanisms such as reduced insulin/IGF-1 pathway or dietary restriction induce autophagy and proteasome activity [86,145,146,147,148], resulting in a delay of polyQ-expanded aggregation and other HD-related changes [17,48].

Further evidence of a link between the age-related decline in proteostasis and HD was provided by studies using induced pluripotent stem cells (iPSCs) from HD patients. These cells opened a new door to a better understanding of the molecular mechanisms underlying HD. As such, iPSCs are an invaluable resource for drug screening to identify novel therapeutic approaches [149]. HD-iPSCs can be terminally differentiated into neurons (including striatal neurons) with HD-related phenotypes such as cumulative risk of death over time and increased vulnerability to stressors [149]. In addition, proteotoxicity induced via oxidative stress or autophagy inhibition leads to increased degeneration in HD neurons compared with controls [149]. Despite these HD-related changes, mutant HTT-expressing neurons present important limitations for disease modeling such as lack of polyQ aggregation and robust neurodegeneration [149,150]. Despite the efforts to detect polyQ-expanded inclusions under different conditions (e.g., cellular stressors, proteasome inhibitors) in neurons from HD-iPSCs, the presence of aggregates has not been observed in these cells [149,150]. The lack of polyQ aggregates in HD neurons could reflect the long period of time before aggregates accumulate in HD, supporting a role of age-related proteostasis dysfunction in this process [149]. Remarkably, HD human neurons do not accumulate detectable mutant HTT inclusions at 12 weeks after transplantation into HD rat models whereas these inclusions could be observed after 33 weeks of transplantation [150]. These findings also suggest a rejuvenation process during the reprogramming process that prevents polyQ aggregation in differentiated neurons until they undergo a demise of proteostasis with age. Indeed, iPSCs exhibit an increased proteostasis network linked with their immortality and ability to generate differentiated cells with an intact proteome [151,152,153]. For instance, iPSCs have increased assembly of the TRiC/CCT complex, a process regulated by intrinsic high levels of the CCT8 subunit [80]. In addition, iPSCs exhibit enhanced expression of PSMD11/RPN6, resulting in up-regulation of proteasome assembly and activity [154]. In somatic tissues, overexpression of CCT8 and PSMD11/RPN-6 induces TRiC/CCT assembly and 26S/30S proteasome assembly, respectively [80,154]. Moreover, overexpression of CCT8 and PSMD11/RPN6 in neuronal cells of HD *C. elegans* models mimics the proteostasis network of immortal iPSCs and reduces polyQ-expanded aggregation [80,154]. Besides intrinsic TRiC/CCT and proteasome assembly, pluripotent stem cells also exhibit autophagy induction during their neural differentiation, supporting the hypothesis of a rejuvenation step to generate “healthy neurons” [155,156].

## 6. Concluding Remarks

In the last two decades, numerous studies have demonstrated the positive impact on HD-related changes induced by mechanisms that can enhance proteostasis or preserve this network during the aging process. Modulation of distinct nodes of the proteostasis network can have distinct effects on mutant HTT to control its toxicity: it can reduce the aggregation or formation of mutant HTT oligomers and also stimulate the degradation of these toxic factors. Moreover, manipulation of proteostasis either genetically or pharmacologically can also compensate the proteostasis collapse induced by mutant HTT expression. Thus, it will be of central importance to define novel regulatory proteostasis pathways. In this paradigm, it will be important to define regulatory pathways that can simultaneously enhance multiple proteostasis nodes or delay the global proteostasis decline characteristic of the aging process. In these lines, iPSCs from HD patients represent an important source of human striatal neurons for discovery of proteostasis regulators in the relevant cells. A potential step towards personalized cell therapy will be genome editing of HD-iPSCs to compensate proteostasis defects in their derived neurons.

## Abbreviations

CMA	Chaperon-Mediated Autophagy
HD	Huntington’s disease
HTT	*Huntingtin*
HSR	Heat-shock response
ER	Endoplasmic reticulum
ERAD	Endoplasmic reticulum-associated degradation
iPSCs	Induced pluripotent stem cells
TRiC/CCT	T-complex protein-1 ring complex/Chaperonin containing TCP1 complex
UPS	Ubiquitin-proteasome system
